# Self-reported problems in medication self-management in older polymedicated patients in general practice

**DOI:** 10.3389/fpubh.2026.1834501

**Published:** 2026-05-19

**Authors:** Anneke Luegering, Tatjana Richter, Albert Lukas, Robert Langner, Stefan Wilm, Thorsten R. Doeppner, Dirk M. Hermann, Helmut Frohnhofen, Carla Stenmanns, Janine Gronewold

**Affiliations:** 1 Hospital Pharmacy, Medical Faculty and University Hospital Düsseldorf, Heinrich Heine University Düsseldorf, Düsseldorf, Germany; 2 Center for Health and Society, Institute of General Practice, Medical Faculty and University Hospital Düsseldorf, Heinrich Heine University Düsseldorf, Düsseldorf, Germany; 3 Department of Orthopedics and Trauma Surgery, Medical Faculty and University Hospital Düsseldorf, Heinrich Heine University Düsseldorf, Düsseldorf, Germany; 4 Department of Geriatrics, St. Martinus Hospital, Düsseldorf, Germany; 5 Institute of Systems Neuroscience, Medical Faculty and University Hospital Düsseldorf, Heinrich Heine University Düsseldorf, Düsseldorf, Germany; 6 Institute of Neuroscience and Medicine (INM-7: Brain and Behavior), Research Center Jülich, Jülich, Germany; 7 Department of Neurology, Klinikum Emden, Emden, Germany; 8 Research Institute for Health Sciences and Technologies (SABITA), Istanbul Medipol University, Istanbul, Türkiye; 9 Department of Anatomy and Cell Biology, Medical University of Varna, Varna, Bulgaria; 10 Department of Neurology, University of Göttingen, Göttingen, Germany; 11 Department of Neurology and Center for Translational Neuro- and Behavioral Sciences (C-TNBS) University Hospital Essen, University Duisburg-Essen, Essen, Germany; 12 Faculty of Health, University Witten Herdecke, Witten, Germany

**Keywords:** medication management capacity, medication self-management, older adults, polypharmacy, self-administration

## Abstract

**Background:**

Polypharmacy is common in the older population and is recognized as a geriatric syndrome. The self-administration of medication is complex and requires cognitive and physical capacities that decrease with age. To avoid medication errors or harm, medication management capacity needs to be thoroughly assessed in routine clinical care. Here, we report on measuring self-reported medication management capacity in older polymedicated patients in primary care in an exploratory study using a questionnaire developed in the “Ability to Self-administer Medication in Non-demented In-hospital Patients” (ABLYMED) study.

**Methods:**

102 patients (≥70 years of age, regularly taking ≥5 different medicines autonomously) from a single general practice in Germany were included from March to November 2024. Patients were interviewed using a questionnaire developed in the ABLYMED study. It consists of 30 questions covering the three domains adherence [16 questions including 5 questions of the Medication Adherence Report Scale (MARS© Professor Rob Horne)], handling (8 questions) and belief/attitude/knowledge (6 questions).

**Results:**

The median (Q1; Q3) age of the study sample was 77 years (74; 84), 39% were male. In the domain adherence, 20% of the patients reported running out of medication and 5% reported mixing up medication, which is very relevant to safety of medication use. Regarding handling problems, eye drops were identified as the most challenging dosage form, with 40% of patients reporting difficulties. Furthermore, 19% of the sample reported handling problems due to problems with opening packages. In the domain belief/attitude/knowledge, only 15% of the patients did not know the rationale behind all of their medications and only 3% did not express a positive perception of their benefits.

**Discussion:**

Our study showed that a high proportion of older patients from a general practice (up to 40%) reported medication handling problems. These results were similar to those observed in the ABLYMED sample of hospital in-patients. Future studies need to concentrate on the implementation of interventions to address patients' self-reported medication management problems.

## Introduction

The prescription of medication represents the most common treatment option for older patients (1). Almost 40% of the older population worldwide is confronted with polypharmacy, defined as the regular use of five or more prescribed medicines (2). When potential benefits of the medication use outweigh its potential harms, polypharmacy is appropriate, otherwise it is inappropriate and problematic (3). The management of polypharmacy is imperative to prevent adverse drug events and medication harm, and to facilitate safe and independent living (4, 5). However, the process of self-administering medication under conditions of polypharmacy places considerable demands on older individuals' cognitive and physical capacities, which decrease with age (6, 7). Physical, cognitive and sensory abilities, as well as motivational and environmental factors can influence medication management capacity. These factors must be considered when prescribing medication to older individuals (8). During routine clinical examinations, inadequate medication management capacity may not be detected because it is not assessed in detail. In addition to the standard clinical evaluation, the application of structured assessments is necessary to gain a deeper understanding of patients' medication management capacity (7, 9). The Lawton Instrumental Activity of Daily Living (IADL) scale, which is an established self-report instrument in the field of geriatric assessment, is designed to evaluate high-level activities of daily living. These activities encompass a range of tasks, including managing medication. The management of medication is, however, only addressed in one question, which may not be sufficient for detecting patients at risk of medication management problems (7, 9, 10). Self-report-based instruments that specifically relate to medication management capacity are also available, but they are not used as frequently as the Lawton IADL scale (4, 8, 11).

As medication management problems, including those relating to handling, continue to be underreported, the “Ability to Self-administer Medication in Non-demented In-hospital Patients” (ABLYMED) study was designed to develop a performance- and self-report-based instrument capable of identifying patients at risk of medication management problems (12). The present analysis focuses on the self-report section of the instrument and presents the results of the ABLYMED questionnaire, which was administered for the first time to a sample of patients attending a general practice (13). The ABLYMED questionnaire consists of self-developed questions as well as the Medication Adherence Report Scale (MARS© Professor Rob Horne) (14–16). The analysis addresses a gap in the literature, as no short questionnaire suitable for general practice patients is currently available (8).

## Material and methods

### Setting and study design

In our cross-sectional single-center observational study we recruited regularly attending patients (patients already included in the database, no new or walk-in patients) from a general practice (internal medicine, located in Troisdorf, Germany, a suburban area between Bonn and Cologne). Patients were included if they were ≥70 years of age and took ≥5 different medicines autonomously. Recruitment took place from March to November 2024. Exclusion criteria were a dementia diagnosis (ICD-10-code F00-F03), legal guardianship, insufficient ability to self-administer medication at home, insufficient ability to communicate, poor vision, agraphia, alexia, palliative condition. We assessed patient characteristics, current medication, medication management capacity via self-report questionnaire, and medication administration performance via evaluation of self-administration of five different placebo dosage forms. The study design complied with the design of the ABLYMED study performed in hospitalized patients (12). In the present analysis, we focus on the questionnaire assessing subjective medication management capacity.

### Questionnaire design and data collection

We assessed clinical and demographic features such as age, sex, current medication including prescribed and non-prescribed medicines, current diseases from medical records, housing situation, Lawton IADL score (10) and Barthel-Index (17) as well as medication management capacity via self-report questionnaire. The questionnaire, designed to evaluate the patient's subjective medication management capacity, was developed in the ABLYMED study via a Delphi process (13). It comprises 30 questions addressing the three domains adherence (MARS, (instrument's range: 5–25, scores < 25 indicating incomplete adherence) (14–16) and self-developed questions), medication handling, and belief/attitude/knowledge about the medication. The questions were self-developed based on a literature review and Delphi process, except for those included in the MARS. The possible response options of the single questions ranged from a binary “yes” or “no” option to a 3- or 5-point Likert scale (13). An evaluation of the 30 questions was conducted in the ABLYMED study by two independent investigators (HF/JG) according to a predetermined scale of relevance to the safety of medication use [“very relevant” (A), “probably relevant” (B), or “less relevant” (C)]. A subject-related dialogue clarified any existing discrepancies. Very relevant to the safety of medication use were the items about handling problems, adherence (assessed via MARS) and the items regarding running out of medications and mixing up of medications.

TR (general practitioner) approached all study patients personally within the context of regular consultations so that patients were continuously included in the study. In a face-to-face interview the questionnaire was administered by TR, who asked the questions and recorded the study patients' responses. Completion of the interview took approximately 10 minutes.

### Ethical considerations

The study was approved by the ethics committee at the Medical Faculty of the Heinrich Heine University Düsseldorf (Reference Number 2021-1435_3). All patients provided written informed consent prior to participating in the study. Due to the nature of primary care practice management, the sample was based on convenience sampling. None of the patients who were approached declined to take part in the study. Data collection took place within the context of regular consultations and was planned in advance to avoid disrupting the practice routine. Data was collected on paper and pseudonymized. The collected data and the pseudonymization list were stored separately.

### Statistical analysis

The frequencies of response options of the single questions were evaluated. For the question addressing handling problems, only patients were considered who had been using the respective dosage form. The analysis of the data was conducted using SPSS 28 for Windows (IBM Corporation, Armonk, NY, United States). As this is a single-center, exploratory study, we report descriptive statistics.

## Results

### Clinical and demographic features

The study comprised a total of 102 patients, who were included from March to November 2024 into the study. The median age (Q1; Q3) was 77 years (74; 84) with a range from 70 years to 96 years. Table 1 describes the study sample.

**Table 1:** Sample description.

Characteristics	Number (%) unless otherwise specified
Median age (Q1; Q3) in years	77 (74; 84)
Men	40 (39)
Women	62 (61)
Median number of medicines (Q1; Q3)	7 (5; 8)
Medical conditions	
Arterial hypertension	89 (87)
Type 2 diabetes mellitus	39 (38)
Hypothyroidism	26 (25)
Housing situation	
Single-person household	44 (43)
Multi-person household	58 (57)
Activities of daily living	
Median Lawton IADL score (10) (Q1; Q3)	8 (6; 8) (Instrument's range: 0–8)
Median Barthel-Index (17) (Q1; Q3)	100 (85; 100) (Instrument's range: 0–100)

### Medication adherence

**Figure 1:**
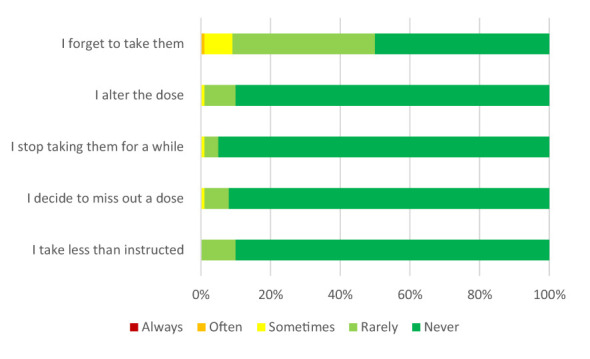
MARS scores. Percentages for each response option are shown for each item.

Table 2 lists the ABLYMED questionnaire items regarding medication adherence and the observed frequencies of each response option per item. Items in bold code the classification of the questions to the designated category A regarding very high relevance to the safety of medication use. In the domain adherence (self-developed questions), 20% of the patients reported running out of medication and 5% reported mixing up medication. These two items are considered very relevant to the safety of medication use. Thirty two percentage of the patients had difficulties with the pharmacy switching companies (generic substitution), which can lead to a different appearance, a different name and a different packaging. In particular, the packaging helped 77% of the study patients to recognize their medicines. Eighty five percent of the study participants tolerated their medication. Eighty nine percent had a complete medication schedule provided by the primary care physician. Adherence assessed via MARS showed that 53% of our study patients did not fully adhere to their medication, defined by a MARS score < 25. Forgetfulness was the predominant cause of non-adherence: 50% of the patients reported to have forgotten to take their medication at least rarely. Figure 1 shows the MARS scores for each item.

**Table 2:** Frequency of self-reported medication management problems as assessed by the ABLYMED questionnaire regarding medication adherence.

Questionnaire item (number of patients who responded to the question)	Number (%)
Adherence, self-developed questions	
Have you ever run out of your medications? (A) (101)	**Yes 20 (20)**
**Have you ever mixed up your medications? (A) (101)**	**Yes 5 (5)**
Do you use any technological aids to remind you to take your medications? (B) (101)	Yes 3 (3)
Do you wish to be reminded to take your medication? (B) (101)	Yes 10 (10)
Does someone remind you to take your medications every day? (B) (101)	Yes 17 (17)
How do you best recognize your medications? (multiple items possible) (B) Appearance (100)	Yes 44 (44)
Name (100)	Yes 24 (24)
Packaging (100)	Yes 77 (77)
Do you have problems with your pharmacy switching companies? (B) (100)	Yes 32 (32)
Do you have a complete medication schedule from your primary care physician? (B) (100)	Yes 89 (89)
Are you tolerating your medication? (B) (101)	Yes 86 (85)
	Moderately well 10 (10)
	No 5 (5)
Adherence, MARS © Professor Rob Horne	
**I forget to take it (A) (101)**	**Always 0 (0)**
	**Often 1 (1)**
	**Sometimes 8 (8)**
	**Rarely 41 (41)**
	**Never 51 (50)**
**I alter the dose (A) (101)**	**Always 0 (0)**
	**Often 0 (0)**
	**Sometimes 1 (1)**
	**Rarely 9 (9)**
	**Never 91 (90)**
**I stop taking it for a while (A) (101)**	**Always 0 (0)**
	**Often 0 (0)**
	**Sometimes 1 (1)**
	**Rarely 4 (24)**
	**Never 96 (95)**
**I miss out a dose (A) (101)**	**Always 0 (0)**
	**Often 0 (0)**
	**Sometimes 1 (1)**
	**Rarely 7 (7)**
	**Never 93 (92)**
**I take less than instructed (A) (101)**	**Always 0 (0)**
	**Often 0 (0)**
	**Sometimes 0 (0)**
	**Rarely 10 (10)**
	**Never 91 (90)**

Bold items = Category, A = Very relevant to safety of medication use, Category B = Probably relevant to safety of medication use, Category C = Less relevant to safety of medication use.

### Medication handling problems

Table 3 lists the ABLYMED questionnaire items regarding medication handling problems and the observed frequencies of each response option per item. Eye drops seemed to be a challenge for older people, with 40% reporting handling problems. For all other dosage forms, the proportion of patients experiencing handling problems was less than 20%, with a range from 19% (handling problems with opening packages) to 5% (handling problems with inhaler device).

**Table 3:** Frequency of self-reported medication management problems as assessed by the ABLYMED questionnaire regarding medication handling problem.

Questionnaire item (number of patients who have been using the respective dosage form)	Number (%)
Do you have problems with…: (A) opening packages? (102)	**Yes 19 (19)**
**Your inhaler? (20)**	**Yes 1 (5)**
**Adhesive bandages? (0)**	**not prescribed**
**Eye drops? (5)**	**Yes 2 (40)**
**Pens? (7)**	**Yes 1 (14)**
**Drops? (6)**	**Yes 1 (17)**
**With dividing tablets? (101)**	**Yes 8 (8)**
Do you need any improper aids to use your medications? (B) (101)	Yes 10 (10)

Bold items = Category, A = Very relevant to safety of medication use, Category B = Probably relevant to safety of medication use, Category C = Less relevant to safety of medication use.

### Beliefs, attitude, and knowledge

In the domain belief/attitude/knowledge about the medication (Table 4), patients appeared to be well informed about their medication: 85% understood the rationale behind their medications, and 75% expressed a positive perception of their benefits.

**Table 4:** Frequency of self-reported medication management problems as assessed by the ABLYMED questionnaire regarding beliefs, attitude and knowledge.

Questionnaire item (number of patients who responded to the question)	Number (%)
Do you feel like your medication changes too often? (B) (96)	Yes 2 (2)
	Moderately 5 (5)
	No 89 (93)
Do you have the impression that you have to take too many medications? (C) (101)	Yes 14 (14)
	In some cases 22 (22)
	No 65 (64)
Do you read the package insert? (C) (101)	Yes 34 (34)
	In some cases 38 (38)
	No 29 (28)
Do you understand the package insert? (C) (75)	Yes 28 (37)
	In some cases 44 (59)
	No 3 (4)
Do you know why you were prescribed your medications? (C) (101)	Yes 86 (85)
	In some cases 15 (15)
	No (0)
Do you think that all medications you have been prescribed help you? (C) (100)	Yes 75 (75)
	In some cases 22 (22)
	No 3 (3)

Bold items = Category, A = Very relevant to safety of medication use, Category B = Probably relevant to safety of medication use, Category C = Less relevant to safety of medication use.

## Discussion

### Comparison with the ABLYMED in-patient study

By assessing medication management capacity via the ABLYMED self-report questionnaire in 102 older, polymedicated patients from a single general practice in 2024 in Germany, we showed that a high proportion (up to 40%) reported medication management problems that are very relevant to the safety of medication use. In particular, handling problems with different dosage forms and running out of medication were the most frequently reported problems.

The ABLYMED questionnaire was originally administered exclusively to an in-patient sample in 2021 and 2022 (13). In contrast, we now used the ABLYMED questionnaire in a general practice sample. All other criteria regarding age (≥70 years) and medication (≥5 different medicines taken autonomously) were the same as in the original ABLYMED study. When comparing the two samples, in-patients were slightly older [median 78 years (Q1; Q3 = 73; 82 years)] than general practice patients [median 77 years (Q1; Q3 = 74;84 years)]. They also self-administered a higher number of medicines [median 9 (Q1; Q3 = 7;12)] compared with general practice patients [median 7 (Q1;Q3 = 5;8)], while the median Lawton IADL score was identical in both samples [median 8 (Q1;Q3 = 5;8) vs. (Q1;Q3 = 6;8)] (18).

A comparison of the frequencies of the individual response options for the questions assessing medication adherence between the in-patient and general practice patient group showed that 20% of the general practice and 11% of the in-patient group reported running out of medication at some point. Five percent of the general practice and 2% of the in-patients group reported mixing up their medication at some point. Concerning adherence, 53% of the general practice patient group and 55% of the in-patient group did not completely adhere to their medication, defined as not achieving the maximum score of 25 (13).

Regarding self-reported medication handling problems, eye drops were the most difficult dosage form for patients from the general practice (40% reported handling problems) as well as for in-patients (43% reported handling problems). Opening packages was challenging for 19% of the general practice patient group and 37% of the in-patient group. In general, both groups reported challenges in handling the various dosage forms. The frequency of these problems varied according to the dosage forms in the two different groups (13).

In the section beliefs, attitude and knowledge of the questionnaire, 7% of the general practice sample criticized frequent changes in medication while 12% did so in the in-patient sample. Regarding package inserts, 72% of the general practice patients reported reading them and 96% understand them at least in some cases. In the in-patient sample, approximately 10% fewer reported reading (61%) and understanding (86%) the package insert.

### General discussion

In our present analysis, we observed that 53% of the study group exhibited non-adherence, defined by a MARS score below 25 points. Gomes et al. assessed adherence in 1,089 polymedicated patients (≥65 years old) in primary care centers in Portugal via a questionnaire [Adherence Treatment Measure (MAT)], observing a non-adherence rate of 47.7%. One major reason for non-adherence was forgetfulness, which was reported by 38.8% of the participants. In our analysis, 50% of the participants reported having forgotten to take their medication at least rarely. In the Portuguese study, 28.9% stated that they had to interrupt their treatment because of running out of medication (1). This phenomenon was reported by 20% of our study patients.

Mehuys et al. (19) assessed patient-reported handling problems with eye drops in community pharmacies in Belgium in a more detailed way. They used a questionnaire comprising ten questions about problems with eye drop instillation such as difficulties with instillation of the drop in the eye, too many drops coming out of the bottle and difficulties with opening the bottle. 39.7% of the 678 study participants (age 68.9 ± 12.4 years) reported at least one type of problem. The most common problem (18.3%) was the instillation of the drop in the eye (19). Another study in a smaller sample confirmed those as well as our findings: Burns et al. (20) examined practical problems associated with the administration of eye drops using a questionnaire completed by 43 outpatients aged ≥75 years. In that study, 12 patients self-administered their eye drops, and 5 (42%) of them reported handling problems (20). The second most prevalent handling problem in our study was that of opening packages with 19% of patients experiencing difficulties. Sormunen and colleagues (21) examined the performance of opening four different types of pharmaceutical packaging in 15 older women aged 69–79 years. The study participants were asked to perform a task with each of the four types of pharmaceutical packaging, and the time taken to complete each task was measured. The results showed that 7% of the participants were unable to open one of the four different types of pharmaceutical packaging (21). Wilbur et al. (22) reported handling problems in 7%−28% of 60 older adults ≥75 years using different types of packaging. Both Sormunen et al. and Wilbur et al. assessed handling problems via performance-based assessments. It is important to note that there may be significant discrepancies between the self-reported abilities and actual performance-based abilities (23, 24).

Krause et al. assessed self-reported medication knowledge and awareness of medication of 100 older (≥70 years) in-patients in Germany. It was established that 52% of the patients reported that their general practitioner contributed most to their medication knowledge. Twelve percent reported consulting the package insert as information sources (25). In our analysis, 34% of the primary care patients reported that they read the package inserts. A bias due to the presence of the general practitioner cannot be ruled out for our study sample and may explain these discrepancies.

### Strengths and limitations

Our present analysis clearly demonstrates the prevalence of subjective problems pertaining to self-administered medication use among older patients. Our study is the first to assess this in detail within a primary care setting in Germany. Nevertheless, several limitations of this exploratory study must be considered. The questionnaire was administered to a moderately sized *ad-hoc* sample of 102 older patients. Patients were recruited consecutively by one general practitioner. Although inclusion criteria were strictly followed, the physician's prior knowledge of the patients may have introduced a selection bias and there might be a social desirability bias since TR conducted the patient interviews herself. Furthermore, we recruited patients from only one general practice in a suburban area, which has a specific patient profile. To substantiate the findings of this study, future research should examine a larger, population-based sample. Despite the limited sample size, our results correspond well with those reported in the current literature reporting results from larger samples.

## Conclusion

The present study utilized an older general practice patient population with polypharmacy and independent medication management to assess subjective medication management problems via the ABLYMED questionnaire. This approach yielded important results for the understanding of medication management problems from the perspective of patients, showing that up to 40% of patients reported medication management problems that are very relevant to the safety of medication use, and more than half of the sample not fully adhering to their medication. It is imperative to gain a comprehensive understanding of patients' medication management issues to implement effective interventions that address these concerns. General practice care facilitates the opportunity to identify patients with medication management problems and to implement targeted interventions due to regular, longitudinal, trusting and comprehensive patient contact. Future studies should focus on the implementation and evaluation of interventions for patients with self-reported medication management problems.

## Data Availability

The datasets analysed for this study are available from the corresponding author on reasonable request.
